# Bovine Rumenotomy1Read at the tenth annual meeting of the Illinois Veterinary Medical and Surgical Association, Decatur, Ill., January 13, 1899.

**Published:** 1899-04

**Authors:** S. H. Swain

**Affiliations:** Decatur, Ill.


### BOVINE RUMENOTOMY.[1]

By S. H. Swain, V.S.,
DECATUR, ILL.

This operation requires considerable skill and dexterity on the part of the operator to perform with nicety and success.

[1] Read at the tenth annual meeting of the Illinois Veterinary Medical and Surgical Association, Decatur, Ill., January 13, 1899.

The indications for the operation are impaction of the rumen with solid food, accompanied with paralysis of the muscles of the rumen, or when it contains a mass of fermenting food with a diffusion of gases.

The preparation for the operation consists in securing at least two or three assistants, who should be instructed in their duties and the securing of the animal; the clipping or shaving of the hair, from the spot selected for the operation; then the cleansing of that region with a solution of bichloride of mercury of the strength of 1 to 500 parts of warm water; next the arrangement and proper cleansing with antiseptics of the necessary instruments, consisting of a knife or scalpel, two or three strong blunt hooks, a vulsellum forceps, and four curved needles, two armed with sutures of silk or linen, one with carbolized catgut, and one with a metallic suture. The instruments should be arranged on a clean tray or other convenient receptacle in the hands of an assistant. Sufficient cleansing solution and clean towels should also be conveniently arranged.

The operator, having decided on the position of his patient during the operation, at once proceeds to secure the animal. If the standing position is decided upon, the animal should be closely and securely tied by the head in a corner, with the right side to the wall; an assistant should stand at the shoulder and one at the hip; one hand of the assistant on the point of the ilium, the other hand on the tail, in order to control that organ. The animal is now ready for operation in the standing position.

Should the recumbent position be chosen the animal should be securely tied to a firm wall or post, with strong head-halter, allowing it about twenty-four inches of liberty, then a strong strap should be carried around the hind limbs above the hocks and crossed between them and buckled close, so as to hold the legs close together; then a rope sufficiently strong and about twelve or fifteen feet in length, with noose on one end, should be looped on the right hind leg below the hock, and carried once and a half around both limbs, the free end being brought between the limbs from in front backward and above the coil around the limbs, so as to form a complete loop on both posterior limbs, the free end of the rope then carried round a post arranged directly in the rear of the animal. The operator directs one assistant at the head, the other assistants on the rope in the rear of the animal. The operator, standing at the left side, directs the assistants at the rope to put on sufficient traction to carry the hind limbs out from under the animal, at the same time the operator gently pushes him over on the right side; then the rope is secured to the post so as to extend the posterior limbs behind the animal; then the anterior portion of the animal should be slightly elevated in order to admit of more freedom of respiration during the operation. In this way the patient is well secured, extended on the right side.

The animal now being properly secured in the position chosen, the operator should proceed as expeditiously as possible with the operation. Assuming a convenient position for operation, and beginning four inches posterior to the last rib and three inches inferior to the bony transverse processes of the lumbar region, he makes an incision six inches in length through the skin and integument, then the peritoneum is divided, next the rumen is opened, beginning a little inferior to the superior margin of the opening in the side; cut through the walls of the rumen a slip about two inches in length, then with the vulsellum forceps take hold of the lips of the incision and elevate sufficiently to adapt the edges of the rumen to the edges of the skin in the external wound; then with the two curved needles, containing strong sutures, as above mentioned, sew the rumen on both sides of the incision to the skin, after the fashion of working a buttonhole, continuing to enlarge the opening and securing the rumen to the skin until the opening is sufficiently large to receive the hand of the operator; then two assistants, one at either extremity of the animal, each armed with a strong blunt hook inserted in the wound, should dilate the opening so as to facilitate the removal of the contents; this being completed to the satisfaction of the operator, the wound should receive attention by being carefully cleansed with suitable antiseptics; next the rumen should be freed from the skin, the edges carefully trimmed and held in position by the vulsellum forceps in the hands of an assistant. The opening is now closed by continuous sutures of catgut, taking care to fold the edges of the wound inside the rumen, so as to adapt the peritoneum. Having completed its closure it may now be returned and the external wound again carefully cleansed and closed by interrupted metallic sutures in the skin; then thoroughly pack the wound and surrounding parts that have been soiled, with powdered boric acid. Treated thus, an abscess is not likely to supervene.

Having completed the operation, make the animal as comfortable as possible, give stimulants and tonics if indicated; allow a restricted and easily digested diet, and sufficient water for several days, keeping a close watch for complications, which should be treated according to indications.

